# Horizontal transfer of a plasmid possessing *mcr-1* marked with a single nucleotide mutation between *Escherichia coli* isolates from community residents

**DOI:** 10.1186/s13104-022-06079-z

**Published:** 2022-06-03

**Authors:** Yoshimasa Yamamoto, Ayano Higashi, Kanoko Ikawa, Hoa Thi Thanh Hoang, Takahiro Yamaguchi, Ryuji Kawahara, Hideki Noguchi, Thang Nam Nguyen, Diep Thi Khong, Hoa Thi Tran

**Affiliations:** 1 United Graduate School of Drug Discovery and Medical Information Sciences, Gifu University, Gifu, Japan; 2 Department of Microbiology, Osaka Institute of Public Health, Osaka, Japan; 3 Joint Support-Center for Data Science Research, Research Organization of Information and Systems, Mishima, Japan; 4 Center for Medical and Pharmaceutical Research and Service, Thai Binh University of Medicine and Pharmacy, Thai Binh, Vietnam

**Keywords:** Colistin resistance, Horizontal gene transfer, *mcr*, *Escherichia coli*

## Abstract

**Objectives:**

The widespread dissemination of phenotypic colistin-resistant (COR) bacteria in the community threatens public health. The horizontal gene transfer of the mobile colistin resistance gene via plasmids is thought to be one of the main mechanisms for dissemination. However, genotypic evidence to prove this in community settings is limited. This study used genome analysis to demonstrate the direct horizontal colistin resistance gene transfer via plasmids in isolates from the community.

**Results:**

A total of 19 isolates of COR *Escherichia coli* from stool specimens of 23 residents from seven households in the Vietnamese community were assessed in this study. The whole-genome sequence data of isolates were acquired using a combination of DNBSEQ short-reads and Nanopore long-read sequencing. Analysis of genomic data was performed using online tools such as Geneious. Analysis of the genomic information of COR *E. coli* isolates revealed that the isolates from two residents of different households had a similar IncP1 plasmid possessing *mcr-1.1*, marked with a single nucleotide mutation at the same position. The study provided direct evidence to prove that *mcr* was horizontally transmitted among bacteria in community residents.

**Supplementary Information:**

The online version contains supplementary material available at 10.1186/s13104-022-06079-z.

## Introduction

The wide dissemination of colistin-resistant (COR) Enterobacteriaceae with a mobile colistin resistance gene, *mcr*, has been reported worldwide, especially in some Asian countries [1–3]. COR bacteria can spread to humans from livestock and animal foods [4, 5]; additionally, a horizontal transfer of plasmids with *mcr* has been reported as an important theoretical mechanism in microbial communities [6]. However, demonstration of horizontal gene transfer requires the identification of plasmids with the same *mcr* in strains isolated from different sources. In this regard, performing such identification is difficult in the community because it is not easy to find a genetically identical plasmid harboring *mcr* in strains isolated from the field due to plasmid alterations during adaptation to different bacterial host conditions. Even if different strains possess the same gene, it is difficult to determine the origin of the gene without a specific mark on the gene. In fact, *mcr* has been found in various plasmid types in COR strains isolated from many sources. Many variants of the *mcr* gene have been reported; however, none have been investigated in association with horizontal gene transfer of the *mcr* gene in community settings.

In this study, we performed sequencing to build whole genomes of COR *E. coli* isolates from community residents in the same area, analyzed genomes using an alignment tool, detected the genetic mark on plasmids, and examined highly homologous sequences for genetic relatedness among strains to obtain direct evidence of horizontal gene transfer of *mcr* via plasmids.

## Main text

### Materials and methods

#### *E. coli* isolates

A total of 19 COR *E. coli* community isolates with *mcr-1* were assessed in this study. All COR *E. coli* isolates were initially obtained from healthy residents of seven households in a rural community of Vietnam in November 2017 [1]. One isolate was obtained from each resident. The characteristics of isolates are listed in Table 1.

**Table 1:**
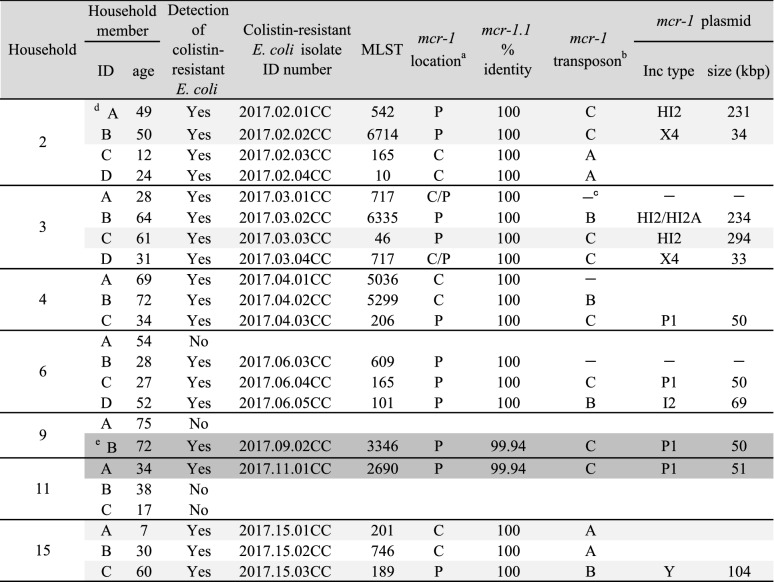
Characteristics of colistin-resistant *E. coli* isolates from household members

^a^*P* plasmid, *C* chromosome

^b^A: IS*Apl1*-*mcr1*-*PAP2*-IS*Apl1*; B: IS*Apl1-mcr1-PAP2*; C: *mcr1-PAP2*

^c^–: not determined

^d^Data of shaded isolates was reported in Yamaguchi et al. [8]

^e^Dark shaded isolates possessed *mcr-1* marked with the single nucleotide mutation

#### DNA extraction and quality control

The genomic DNA of isolates was extracted using NucleoBond HMW DNA (MACHEREY–NAGEL, Düren, Germany) in accordance with the manufacturer’s instructions to obtain high molecular weight DNA samples. The DNA was quantified using Qubit Double-Stranded DNA High-Sensitivity Assay Kits (Thermo Fisher Scientific, Waltham, MA, USA) and qualitatively analyzed using NanoDrop (Thermo Fisher Scientific). Nanodrop A260/280 and A260/230 ratios of the extracted DNA samples were within the range required by the manufacture’s instructions.

#### Library preparation and sequencing

Whole-genome sequencing was performed using the DNBSEQ-G400RS (MGI Tech, Shenzhen, China) and MinION Mk1C sequencer (Oxford Nanopore Technologies, London, UK). Short-read sequencing using DNBSEQ was performed by a commercial vendor (Genome-Lead Co., Kagawa, Japan) with a DNBSEQ-G400RS High-throughput Sequencing Set (MGI Tec). For long-read sequencing using MinION, high molecular weight DNA of each isolate was tagmented using the Rapid Barcoding Kit (Oxford Nanopore Technologies); then, all bar-coded samples were pooled and sequencing adapters were attached to prepare a library. The MinION flow cell (R9.4.1) was primed and loaded with the prepared library for a 48-h run on MinION Mk1C. The read quality was verified using NanoPlot (http://nanoplot.bioinf.be/). The read length N50 of each run was 10.35K to 14.62K bases (longest read: 160 Kb). The sequencing run generated 125,267 to 342,484 reads. Total bases aligned was 357 to 990 million bp.

#### Bioinformatic analysis

The de novo hybrid assembly of short and long base sequence reads was performed using Unicycler 0.4.8 with default settings through a supercomputer system [7]. Briefly, SPAdes v3.13.1 (with the maximum k-mer 127), miniasm, Racon v.1.4.3, bowtie2 v.2.3.5, SAMtools v.1.9, and Pilon v.1.23 in Unicycler were used to assembly and polish the complete bacterial genome (Additional file 1). The complete genomes were obtained from 14 isolates in this study (Table 1).

#### Annotations

The complete genomes were annotated by uploading the assembled FATSA files to DFAST (https://dfast.ddbj.nig.ac.jp/dfc/) and double-checking using ResFinder 4.1 (https://cge.cbs.dtu.dk/services/ResFinder/), applying a 90% identify threshold and a minimum overlapping length of 60%.

#### Alignments and mutation detection

Genome analysis was performed using Geneious Prime 2021.2 software (https://www.geneious.com), especially to find a single mutation position on the plasmid. Highly homologous complete plasmid sequences available in the National Center for Biotechnology Information database were compared with the plasmid sequences using Easyfig 2.2.2 (https://mjsull.github.io/Easyfig/).

#### Phylogenetic tree generation

Multilocus sequence typing (MLST) phylogenetic tree analysis of the isolates was performed using MEGA 11 to analyze the genetic relatedness among the strains (https://www.megasoftware.net/). A phylogenetic tree was generated using the maximum likelihood method, the Tamura-Nei model with a bootstrap test of 1000 replicates, and other parameters such as gamma distribution of rates among sites, 5 discrete gamma categories, and 32 threads; thus, the initial tree was generated automatically (by default).

#### Accession numbers

The complete genome sequences of COR *E. coli* isolates 2017.04.03CC, 2017.06.04CC, 2017.09.02CC, and 2017.11.01CC were deposited in DDBJ/GenBank under the accession numbers AP025205-AP025222 (Additional file 2).

### Results and discussion

The studied isolates were collected from residents in a previous study [1]. COR bacteria are highly prevalent not only in residents but also in backyard livestock in the area [1, 3]. In this study, 19 *mcr-1*-positive COR *E. coli* strains isolated from the stool specimens of 23 members belonging to seven households were assessed to clarify the genetic context of *mcr-1*; that is, we further expanded a previous study [8] to a detailed genome analysis with the acquisition and analysis of genome sequences of isolates.

**Fig. 1:**
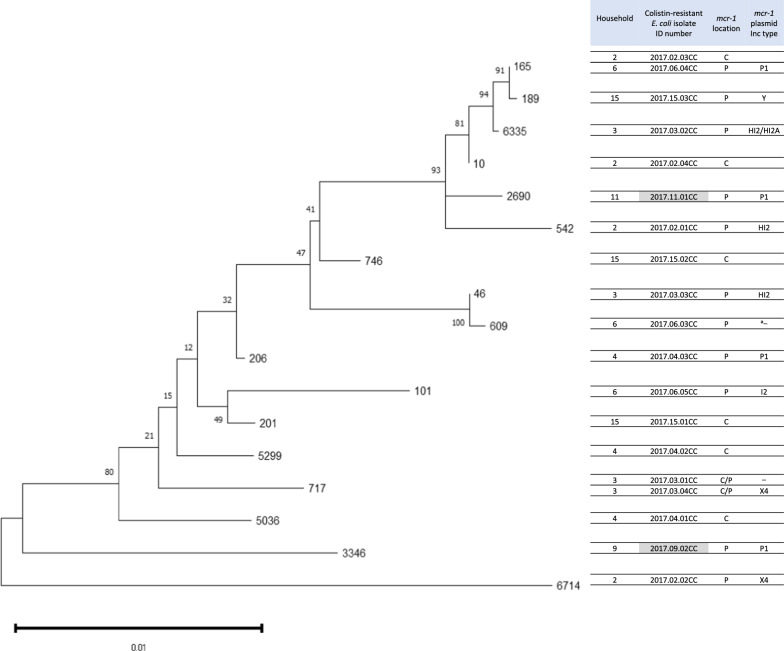
MLST phylogenetic tree of colistin-resistant *E. coli* isolates obtained from household members. Phylogenetic tree generated using the maximum likelihood method and Tamura-Nei model with a bootstrap test of 1000 replicates using MEGA 11. Values at the nodes represent bootstrap support values. The scale bar represents nucleotide substitutions per site. Shaded isolates possess *mcr-1.1* marked with the same single nucleotide mutation. ^a^Unknown

**Fig. 2:**
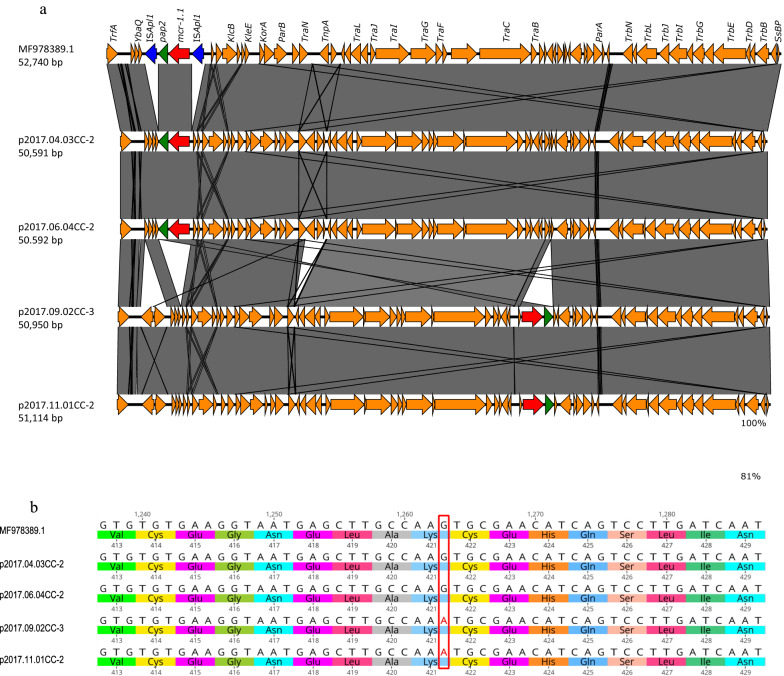
**a** Comparison of the genetic context of *mcr-1.1* in the plasmids of isolates. Genetic plasmid structures containing the *mcr-1.1* gene in IncP1 plasmids of four isolates are shown with a reference IncP1 plasmid (MF978389.1). **b** A single nucleotide mutation site on *mcr-1.1* of p2017.09.02CC-3 and p2017.11.01CC-2 with a reference *mcr-1.1* of MF978389.1. The square indicates the mutation site

As shown in Table 1, members from all households with COR *E. coli* containing *mcr-1* are reported. *mcr-1* was located in the plasmids, chromosomes, or both. Particularly, the prevalence of *mcr-1* on the chromosome was high in eight (42%) out of 19 COR *E. coli* isolates. This high chromosomal prevalence is consistent with a previous report [8]. Two isolates carried *mcr-1* on both chromosomes and plasmids. The *mcr-1*-carrying plasmid analyzed in this study showed Inc-type diversity, as previously reported [9]. Thus, the locations of *mcr-1* and *mcr-1*-plasmids were diverse, even for isolates from households in a limited area. Additionally, phylogenetic analysis of these isolates revealed no spreading of a particular clone (Fig. 1). These results do not directly support the interbacterial transmission of plasmids with *mcr-1*, which has been considered one of the prevalent theoretical mechanisms in the community of COR bacteria. However, a detailed examination of the *mcr-1*-carrying plasmid in this study revealed that IncP1 plasmids of similar size and constituent genes, but not identical components, were present in isolates from members of four households (Fig. 2a).

Further examination of the *mcr-1* gene showed that the gene was 100% identical to *mcr-1.1* in all isolates, including IncP1 plasmids-*mcr-1*, except for two isolates, 2017.09.02CC and 2017.11.01CC. These two isolates possessed IncP1-type plasmids, which were approximately similar in size, 50,950 bp and 51,114 bp, respectively, and had the same constituent genes and *mcr-1.1*, which had a single nucleotide mutation at the 1263rd position from guanine to adenine (Fig. 2b). This mutation was confirmed to be consistent with a read of 6500 (p2017.09.02CC-3) and 9640 (p2017.11.01CC-2) on short read remapping with 99.6% (p2017.09.02CC-3) and 99.0% (p2017.11.01CC-2) of the allele frequency. The mutation was a silent mutation that did not alter the amino acids. The colistin minimum inhibitory concentrations of 2017.09.02CC and 2017.11.01CC were 16 μg/mL and 8 μg/mL, respectively, similar to those of other isolates [1]. This indicated that the mutation did not affect colistin resistance.

As the bacterial mutation rates are typically very low, ranging from one in ten million to one in a billion base substitutions per nucleotide per generation [10], the occurrence of a similar single mutation in the same position among strains in a narrow community should be rare. Therefore, the finding of the same single nucleotide mutation of *mcr* among the strains isolated from different sources in the field is an important finding that indicates *mcr* is transferred between strains in the field, even if the number of isolates tested is limited.

Investigation of plasmids with the same mutation on *mcr-1.1* in different isolates indicated a horizontal transmission of plasmids between bacteria. The MLST values of these isolates were 3346 and 2690 (Table 1). The analysis of the genetic relatedness of these MLSTs showed that the isolates were phylogenetically different (Fig. 1). This indicates that these isolates are not derived from the same clone with the plasmid; that is, the horizontal transfer of plasmid with *mcr* occurs from bacteria to bacteria in the community.

To conclude, the results of this study provided evidence that the resistance gene, *mcr,* was horizontally transmitted between bacteria via plasmids in the community.

## Limitation

The conclusion is based on the results of genome analysis of the isolates from the community. In addition, since the number of strains examined was limited, it is necessary to conduct further studies with larger numbers of strains to obtain generalizable results.

## Supplementary Information

**Additional file 1.** Details of an example of sequence assembly using Unicycler in this study.

**Additional file 2.** Additional data of the complete genome of colistin-resistant *E. coli* isolates.

## Data Availability

Data sets used and/or analyzed in this study are available from the corresponding author on reasonable request.

## Abbreviations

COR: Colistin-resistant
MLST: Multilocus sequence typing
