# Supplementary material for: Horizontal transfer of a plasmid possessing mcr-1 marked with a single nucleotide mutation between Escherichia coli isolates from community residents
Source: BMC Res Notes. 2022 Jun 3;15:196. doi: 10.1186/s13104-022-06079-z (PMC9166650; doi:10.1186/s13104-022-06079-z)
Supplement: Supplementary file 1 — Additional file 1. Details of an example of sequence assembly using Unicycler in this study. [file 13104_2022_6079_MOESM1_ESM.docx]

**Additional file 1**

Details of an example of sequence assembly using Unicycler in this study

Unicycler performs a hybrid assembly. It first use SPAdes to make a short-read assembly graph, and then it uses various methods to scaffold that graph with the long reads.

Unicycler version: v0.4.8

Using 8 threads

Program Version

spades.py 3.13.1

racon 1.4.3

makeblastdb 2.9.0+

tblastn 2.9.0+

bowtie2-build 2.3.5

bowtie2 2.3.5

samtools 1.9

java 11.0.1

pilon 1.23

--------------------------------------------------

Unicycler uses the SPAdes read error correction module to reduce the number of errors in the short read before SPAdes assembly. This can make the assembly faster and simplify the assembly graph structure.

-------------------------------------------------------

Unicycler chooses a k-mer range for SPAdes based on the length of the input reads. It uses a wide range of many k-mer sizes to maximise the chance of finding an ideal assembly.

SPAdes maximum k-mer: 127

Median read length: 149

K-mer range: 27, 47, 63, 77, 89, 99, 107, 115, 121, 127

SPAdes assemblies

---------------------------------------

Unicycler now uses SPAdes to assemble the short reads. It scores the assembly graph for each k-mer using the number of contigs (fewer is better) and the number of dead ends (fewer is better). The score function is 1/(c*(d+2)), where c is the contig count and d is the dead end count.

Determining graph multiplicity

----------------------------------------------------

Multiplicity is the number of times a sequence occurs in the underlying sequence. Single-copy contigs (those with a multiplicity of one, occurring only once in the underlying sequence) are particularly useful.

Cleaning graph

------------------------------------

Unicycler now performs various cleaning procedures on the graph to remove overlaps and simplify the graph structure. The end result is a graph ready for bridging.

Graph overlaps removed

Unicycler now selects a set of anchor contigs from the single-copy contigs. These are the contigs which are connected via bridges to form the final assembly.

Creating SPAdes contig bridges

----------------------------------------------------

SPAdes uses paired-end information to perform repeat resolution (RR) and produce contigs from the assembly graph. SPAdes saves the graph paths corresponding to these contigs in the contigs.paths file. When one of these paths contains two or more anchor contigs, Unicycler can create a bridge from the path.

Creating loop unrolling bridges

-----------------------------------------------------

When a SPAdes contig path connects an anchor contig with the middle contig of a simple loop, Unicycler concludes that the sequences are contiguous (i.e. the loop is not a separate piece of DNA). It then uses the read depth of the middle and repeat contigs to guess the number of times to traverse the loop and makes a bridge.

Loading reads

-----------------------------------

Assembling contigs and long reads with miniasm

--------------------------------------------------------------------

Unicycler uses miniasm to construct a string graph assembly using both the short read contigs and the long reads. It then use the resulting string graph to produce bridges between contigs. This method requires decent coverage of long reads and therefore may not be fruitful if long reads are sparse. However, it does not rely on the short read assembly graph having good connectivity and is able to bridge an assembly graph even when it contains many dead ends.

Unicycler uses two types of "reads" as assembly input: anchor contigs from the short-read assembly and actual long reads which overlap two or more of these contigs. It then assembles them with miniasm.

Aligning long reads to graph using minimap

Finding overlaps with minimap...

success

5,958,201 overlaps

Assembling reads with miniasm...

success

952 segments, 1,574 links

Merging segments into unitigs:

5 circular unitigs

538 linear unitigs

total size = 7,407,140 bp

Polishing miniasm assembly with Racon

-----------------------------------------------------------

Unicycler now uses Racon to polish the miniasm assembly. It does multiple rounds of polishing to get the best consensus.
Circular unitigs are rotated between rounds such that all parts (including the ends) are polished well.

Contigs in the short-read assembly graph which end in dead ends may contain bogus sequence near the dead end. Unicycler therefore uses the read clipping values from the miniasm assembly to trim these dead ends to only the parts which aligned well to long reads.

No dead ends required trimming.

Unicycler now places the single copy contigs back into the unitig graph. This serves two purposes: a) it replaces long read assembly sequences (which may be error prone) with Illumina assembly sequence (which is probably quite accurate), improving the assembly quality, and b) it defines inter-contig sequences for use in building bridges.

Creating miniasm/Racon bridges

----------------------------------------------------

Now that the miniasm/Racon string graph is complete, Unicycler uses it to build bridges between anchor segments.

Creating simple long read bridges

-------------------------------------------------------

Unicycler uses long read alignments (from minimap) to resolve simple repeat structures in the graph. This takes care of some "low-hanging fruit" of the graph simplification.

Aligning long reads to graph using minimap

Two-way junctions are defined as cases where two graph contigs (A and B) join together (C) and then split apart again (D and E). This usually represents a simple 2-copy repeat, and there are two possible options for its resolution:

(A->C->D and B->C->E) or (A->C->E and B->C->D). Each read which spans such a junction gets to "vote" for option 1, option 2 or neither.

Unicycler creates a bridge at each junction for the most voted for option.

Simple loops are parts of the graph where two contigs (A and B) are connected via a repeat (C) which loops back to itself (via D). It is possible to traverse the loop zero times (A->C->B), one time (A->C->D->C->B), two times (A->C->D->C->D->C->B), etc.

Long reads which span the loop inform which is the correct number of times through. In this step, such reads are found and each is aligned against alternative loop counts. A reads casts its "vote" for the loop count it agrees best with, and Unicycler creates a bridge using the most voted for count.

Determining low score threshold

-----------------------------------------------------

Before conducting semi-global alignment of the long reads to the assembly graph, Unicycler must determine a minimum alignment score threshold such that nonsense alignments are excluded. To choose a threshold automatically, it examines alignments between random sequences and selects a score a few standard deviations above the mean.

Automatically choosing a threshold using random alignment scores.

------------------------------------------------

Unicycler uses the long read alignments to produce bridges between anchor segments. These bridges can be formed using as few as one long read, giving Unicycler the ability to bridge the graph even when long-read depth is low.

Applying bridges

--------------------------------------

Unicycler now applies to the graph in decreasing order of quality. This ensures that when multiple, contradictory bridges exist,

--------------------------------------------

The assembly is now mostly finished and no more structural changes are made. Ideally the assembly graph should now have one contig per replicon and no erroneous contigs (i.e a complete assembly). If there are more contigs, then the assembly is not complete.

Polishing assembly with Pilon

---------------------------------------------------

Unicycler now conducts multiple rounds of Pilon in an attempt to repair any remaining small-scale errors with the assembly.

--------------------------------------------------

Any completed circular contigs (i.e. single contigs which have one link connecting end to start) can have their start position changed without altering the sequence. For consistency, Unicycler now searches for a starting gene (dnaA or repA) in each such contig, and if one is found, the contig is rotated to start with that gene on the forward strand.

Assembly complete

---------------------------------------
