# Supplementary material for: Horizontal transfer of a plasmid possessing mcr-1 marked with a single nucleotide mutation between Escherichia coli isolates from community residents
Source: BMC Res Notes. 2022 Jun 3;15:196. doi: 10.1186/s13104-022-06079-z (PMC9166650; doi:10.1186/s13104-022-06079-z)
Supplement: Supplementary file 2 — Additional file 2. Additional data of the complete genome of colistin-resistant E. coli isolates. [file 13104_2022_6079_MOESM2_ESM.docx]

Additional file 2

Additional data of the complete genome of colistin-resistant *E. coli* isolates

| Strain name | Genome size (bp) | GC content (%) | Genome structure (circle) | Length (bp) | Chromosome/ Plasmid name | DDBJ accession number |
| --- | --- | --- | --- | --- | --- | --- |
| 2017.04.03CC | 4,705,884 | 50.8 | Chromosome | 4,560,591 | 2017.04.03CC DNA | AP025205.1 |
|  |  |  | Plasmid 1 | 92,260 | p20170403CC-1 | AP025206.1 |
|  |  |  | ^a^ Plasmid 2 | 50,591 | p20170403CC-2 | AP025207.1 |
|  |  |  | Plasmid 3 | 2,442 | p20170403CC-3 | AP025208.1 |
| 2017.06.04CC | 4,896,524 | 50.8 | Chromosome | 4,623,081 | 2017.06.04CC DNA | AP025209.1 |
|  |  |  | Plasmid 1 | 216,038 | p2017.06.04CC-1 | AP025210.1 |
|  |  |  | Plasmid 2 | 50,592 | p2017.06.04CC-2 | AP025211.1 |
|  |  |  | Plasmid 3 | 4,371 | p2017.06.04CC-3 | AP025212.1 |
|  |  |  | Plasmid 4 | 2,442 | p2017.06.04CC-4 | AP025213.1 |
| 2017.09.02CC | 4,952,248 | 50.7 | Chromosome | 4,702,893 | 2017.09.02CC DNA | AP025214.1 |
|  |  |  | Plasmid 1 | 110,874 | p2017.09.02CC-1 | AP025215.1 |
|  |  |  | Plasmid 2 | 83,929 | p2017.09.02CC-2 | AP025216.1 |
|  |  |  | Plasmid 3 | 50,950 | p2017.09.02CC-3 | AP025217.1 |
|  |  |  | Plasmid 4 | 2,137 | p2017.09.02CC-4 | AP025218.1 |
|  |  |  | Plasmid 5 | 1,465 | p2017.09.02CC-5 | AP025219.1 |
| 2017.11.01CC | 4,915,772 | 50.6 | Chromosome | 4,733,210 | 2017.11.01CC DNA | AP025220.1 |
|  |  |  | Plasmid 1 | 129,419 | p2017.11.01CC-1 | AP025221.1 |
|  |  |  | Plasmid 2 | 51,114 | p2017.11.01CC-2 | AP025222.1 |
|  |  |  | Plasmid 3 | 2,029 | p2017.11.01CC-3 | AP025223.1 |

^a^, Shaded plasmids possessed *mcr-1.*
